# Shared functional connectivity between the dorso-medial and dorso-ventral streams in macaques

**DOI:** 10.1038/s41598-020-75219-x

**Published:** 2020-10-29

**Authors:** R. Stefan Greulich, Ramina Adam, Stefan Everling, Hansjörg Scherberger

**Affiliations:** 1 Deutsches Primatenzentrum GmbH, Kellnerweg 4, 37077 Göttingen, Germany; 2 Robarts Research Institute, University of Western Ontario, London, Canada; 3 Graduate Program in Neuroscience, University of Western Ontario, London, Canada; 4 Department of Physiology and Pharmacology, University of Western Ontario, London, Canada; 5 Faculty of Biology and Psychology, University of Goettingen, Göttingen, Germany

**Keywords:** Neuroscience, Motor control, Sensorimotor processing, Sensory processing

## Abstract

Manipulation of an object requires us to transport our hand towards the object (reach) and close our digits around that object (grasp). In current models, reach-related information is propagated in the dorso-medial stream from posterior parietal area V6A to medial intraparietal area, dorsal premotor cortex, and primary motor cortex. Grasp-related information is processed in the dorso-ventral stream from the anterior intraparietal area to ventral premotor cortex and the hand area of primary motor cortex. However, recent studies have cast doubt on the validity of this separation in separate processing streams. We investigated in 10 male rhesus macaques the whole-brain functional connectivity of these areas using resting state fMRI at 7-T. Although we found a clear separation between dorso-medial and dorso-ventral network connectivity in support of the two-stream hypothesis, we also found evidence of shared connectivity between these networks. The dorso-ventral network was distinctly correlated with high-order somatosensory areas and feeding related areas, whereas the dorso-medial network with visual areas and trunk/hindlimb motor areas. Shared connectivity was found in the superior frontal and precentral gyrus, central sulcus, intraparietal sulcus, precuneus, and insular cortex. These results suggest that while sensorimotor processing streams are functionally separated, they can access information through shared areas.

## Introduction

The two-stream hypothesis regarding hand and arm motor actions has been discussed since the early 1980s^1^ (for review see^2^). It postulates that information processing for reach and grasp actions are implemented in two distinct cortical streams, in the dorso-ventral stream for grasp processing and in the dorso-medial stream for reaching (for review see^3^). The dorso-medial pathway extends from the primary visual cortex over V6A, to the medial intraparietal area (MIP) and to the dorsal premotor cortex (also known as area F2), while the dorso-ventral pathway goes over the anterior intraparietal area (AIP) towards the ventral premotor cortex (in particular: area F5)^4^ and the primary motor cortex (M1), which is well supported by tracer studies^5–14^.

There is direct causal evidence for the separation of both circuits in the form of chemically or magnetically induced lesions in both humans and non-human primates. For example, inactivation of area F5 and AIP is associated with strong deficits on grasping, but no effect on reaching movements, both in macaques^15,16^ and humans^17,18^. In contrast, damage to MIP and V6A, which are often summarized as the parietal reach region PRR^19^, leads to a condition known as optic ataxia that comprises reach deficits as well as minor grasp impairments that are likely a consequence of the patient’s reach uncertainty^20^. Finally, electrophysiological recording experiments in non-human primates confirmed specialized functions of these areas consistent with the two-stream hypothesis^6,21–26^.

However, some studies have found grasp-related activity in traditional reach-related areas and reach-related activity in traditional grasp-related areas, contradicting the notions of a strict separation between both streams^27,28^. Activity related to hand orientation was found in F2^29^ and in V6A^30^, both areas part of the reach-related dorso-medial stream, while reach activity was found in F5^31^, an area part of the grasp-related dorso-ventral stream. Other studies added support for these findings^32–36^, warranting further investigation into the separation between reaching and grasping processes in the brain.

We aimed to resolve whether grasping and reaching functions recruited two distinct functional cortical networks and employed a functional connectivity analysis of resting state functional magnetic resonance imaging (rs-fMRI) data. Unlike tracer studies, rs-fMRI is not limited to monosynaptic connections, but rather allows for the added identification of functionally connected areas through polysynaptic connections. Correlation between areas gives a measurement of how strong they are functionally interlinked^37^. Paired with modern cluster detection^38^, this approach allows us to discern whether the cortical areas F2, F5, M1, AIP, V6A and MIP belong to one or multiple networks. Also, unlike electrophysiological experiments, it allows us to study the whole brain and explore the extension of the resulting networks. We analyzed rs-fMRI data in a population of 10 lightly anesthetized macaque monkeys with seeds placed in six cortical areas in the dorso-medial and the dorso-ventral stream. We show that both processing streams form clearly separated functional networks, however, there are specific areas to which both networks are connected, suggesting a possible communication link between the dorso-medial and dorso-ventral network.

## Materials and methods

Resting state fMRI data was collected from 12 male adult rhesus macaque monkeys (*Macaca mulatta*). Of these, two animals were excluded, one because of a susceptibility artifact over the right parietal lobe and one because of an abnormally shaped central sulcus. The remaining 10 animals had a body weight of 6.1–11.8 kg (mean: 8.1, std: 1.6) and were 5–10 years of age (mean; 6.2, std: 1.7). Animal care and experimental procedures followed the guidelines of the Canadian Council on Animal Care policy on experimental animals. All procedures were approved by the Institutional Animal Care Committee of the University of Western Ontario (Animal Use Protocol Number 2008-125), and all animal experiments were conducted there.

### Anesthesia

Animals were first sedated with 0.1–0.2 mg/kg acepromazine, followed by 7.5 mg/kg ketamine hydrochloride by intramuscular injection, before anesthesia was induced with 2.5 mg/kg propofol via an intravenous catheter in the saphenous vein. Anesthesia was maintained with 1–2% isoflurane with oxygen (1.5–2 l/min) through endotracheal intubation, which was reduced to 1% during resting-state functional imaging. Heart rate, O_2_-saturation, respiration rate and respiratory CO_2_ levels were continuously monitored.

### Data acquisition and preprocessing

Macaque monkeys were scanned in a 7 T Scanner (Siemens, Erlangen, Germany) with a 40 cm gradient coil with field strength of 80 mT/m. A custom build 24-channel phased array head-coil^39^ was used to collect 2-dimensional multi-band T2* weighted EPI images (TR = 1000 ms, TE = 18 ms, flip angle = 40°, 42 slices, resolution 1 × 1 × 1.1 mm, FOV 96 × 96 mm, and matrix size 96 × 96). In total 4 runs of 600 functional volumes were recorded sequentially in one session. Furthermore, standard T1-weighted anatomical images were acquired in the same orientation with 0.5-mm isotropic resolution.

Data was preprocessed with the software package FSL (fMRI Software Library: https://www.fmrib.ox.ac.uk). Functional images were corrected for motion and image acquisition timing. High pass filtering was implemented by subtracting a Gaussian least-square straight-line fit (sd: 100 s) and subsequent low-pass filtering with Gaussian smoothing (sd: 2.8 s). Brain extraction was done with the Brain Extraction Tool and the BrainSuite toolbox^40^. Average EPI images were realigned to the anatomical scans and both co-registered with the standard F99 atlas^41^. Finally, functional images were spatially smoothed (Gaussian filter, FWHM: 3 mm). Further details of the scanning protocol and data preprocessing are included in^42^. Unless stated otherwise, this publication follows the parcellation of the macaque cortex as introduced by^43^.

### Experimental design and statistical analysis

All analyses were based on data from 10 macaque monkeys, as detailed above. Statistical procedures were performed on individual subjects as well as across the subject population, as described in the following sections.

### Seed-based correlation analysis

**Figure 1:**
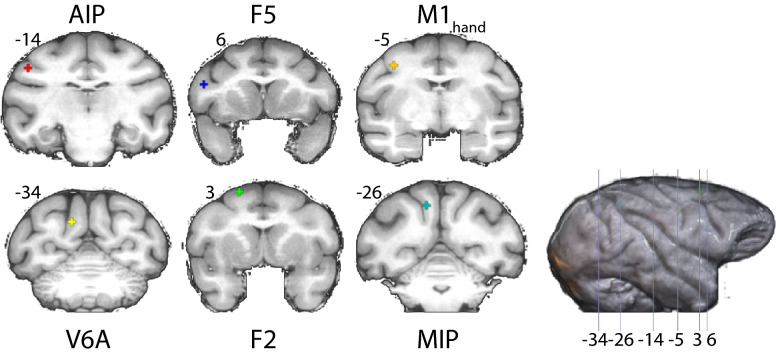
Placement of the seeds in the F99 brain template^45^. Top row, left to right: AIP, F5 and M1_hand_. Bottom row, left to right: V6A, F2 and MIP. Y coordinates of the sections are given relative to the F99 reference frame^45^. Sections rendered with MRIcoGL ver. 1.2.20181114 + (https://www.mccauslandcenter.sc.edu/mricrogl/).

A seed-based correlation analysis was performed with extracted time series from six individual seeds in both hemispheres. All seeds were placed using the Saleem and Logothetis atlas as a reference^44^ in the F99 brain template^45^ (Fig. 1). Seeds in AIP, F5 and M1_hand_ were positioned according to the location of implants in^46^ as guidance to locations with known involvement in grasp planning and execution. Further seeds were placed in V6A and MIP according to locations provided by^34^ and^19^, respectively. F2 seed was placed according to injection site reported in^8^. See supplemental methods for a more extensive description of the seed locations and supplemental Table S1 for the precise coordinates. Seeds had a radius of 1 mm and encompassed 7 voxels. The minimal distance between seeds was between V6A and MIP with 10 mm. Each brain was normalized to the F99 template with FSL, eliminating the need of individual seed placement.

Time series for each seed, cerebrospinal fluid (CSF) and white matter (WM) were extracted for each monkey and run. We calculated whole-brain correlations for each monkey with a general linear model with CSF and WM included as regressor of noninterest. The resulting correlation maps for each animal were then included in a population analysis for each seed individually calculated and modeled independently with a mixed effects model (threshold: p = 0.05, voxel-wise corrected for multiple comparison with GRF-theory-based maximum height thresholding as implemented in FSL).

### Modularity analysis between seeds

For the between seed comparison, correlation coefficients between the extracted time series were calculated and averaged over runs in each individual subject. Mean and variance across individuals was reported in the correlation matrix. These values were tested for significant difference from zero with a two-sided t test with Bonferroni correction for multiple testing. To test whether connections separate into distinct networks, we assessed the optimal modularity^38^, as implemented in the brain connectivity toolbox^47^, and tested for significance (p < 0.05) against the modularity of 1,000,000 randomly shuffled, symmetrical connectivity matrices. Optimal modularity is especially well suited for this research question as the algorithm stops if a network is considered indivisible. It therefore does return an unbiased division into one or more networks^38^.

### Conjunction analysis between seeds

Since all cortical areas show neural correlation with a grasp process^48^, a simple correlation analysis over all seeds together is not applicable since the resulting contrast vectors would not be orthogonal and a simple addition would lead to exaggerated results. Therefore, whenever correlation maps of a network, detected by the modularity analysis, were combined, we used a much stricter approach: only voxels that were significant in each individual seed map (FDR corrected z-score > 2.3) were included in the conjunction map. The results from the individual seed map were converted to a binary mask with a voxel mapped to 1 if it was significantly correlated with the seed or 0 otherwise. Those binary maps were then summed according to the detected networks in the modularity analysis. A similar method, reporting voxels with 2 out of 3 significant tests, was used in^49^. The result is then mapped onto the brain with each voxel value representing in how many seed maps the voxel is significant. We considered only voxels, which were significantly correlated towards all the seeds of a network, to be part of said network.

## Results

Using rs-fMRI of 10 lightly anesthetized macaque monkeys, we obtained connectivity maps by placing seeds in each of the cortical areas AIP, M1_hand_ and F5 of the dorso-ventral stream as well as in F2, V6A and MIP of the dorso-medial stream. For each animal, four rs-fMRI runs were averaged and results from all animals were combined in a multi-effect analysis (see Materials and Methods). In the following section, we first report connectivity maps for individual seed areas and then take a more global approach by generating maps from multiple seeds.

Results from seeds placed in the left or right hemisphere were largely symmetrical. We therefore report only functional connectivity results of the left seeds in both hemispheres. For the results from the seeds in the right hemisphere see Figure S1 and S2 in the supplementary material. Furthermore, since the RF coil used here is known to have a low signal-to-noise ratio in the cerebellum and brainstem, we only report results from cortical and subcortical areas, even though some significant correlations were also found in the cerebellum. Significant results in the midbrain are reported, but due to the low signal, they should be interpreted with caution.

### Functional connectivity map of AIP

**Figure 2:**
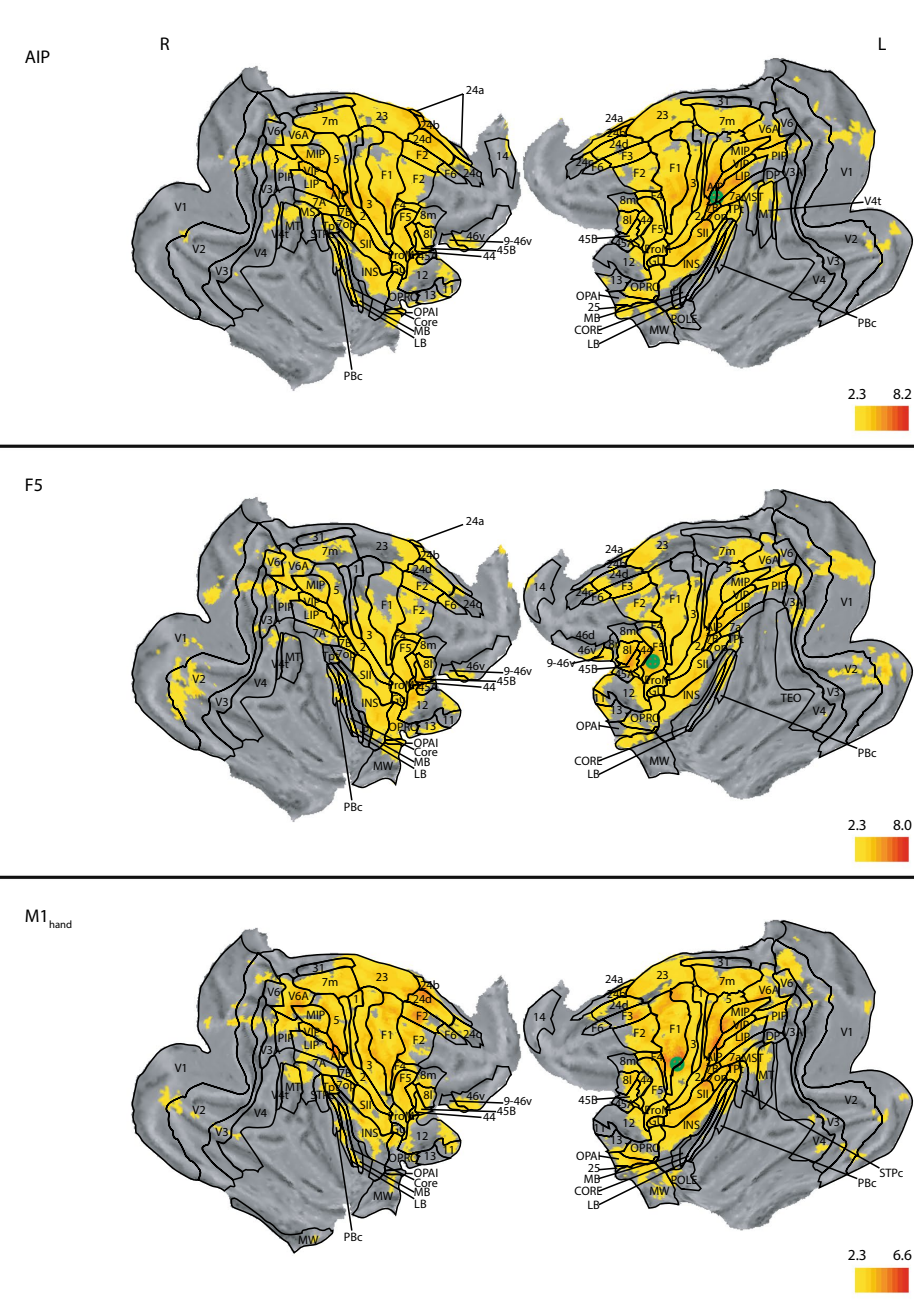
Whole brain correlation of the AIP, F5 and M1_hand_ seeds, projected onto the cortical surface. z-statistic maps are FDR corrected and thresholded according to the z-score color bar. Please note that each seed has a different z-score scaling for the projection. Seed placement is marked by the green marker on the left hemisphere. Since the analysis was done on the 3D dataset, the seed placement marking is approximated. All surface renderings (flat maps) were done with CARET v5.65 (https://brainvis.wustl.edu/wiki/index.php/Caret:About)^88^. Cortical area labeling and borders according to^43^.

The connectivity map of left AIP covers large portions of the parietal lobe, including posterior regions up to the superior temporal sulcus, intraparietal sulcus and the parieto-occipital sulcus (Fig. 2, top panels). In anterior regions, the main correlation cluster reaches up to the arcuate sulcus; however, the cingulate cortex also shows correlation. Additionally, there is correlation in area 13 and lateral area 12. Correlations peak in the cingulate sulcus, the anterior part of the intraparietal sulcus and the middle part of the central sulcus. All these correlations are symmetrical across the left and right hemisphere. Asymmetries appeared in the activation of the superior arcuate sulcus, with a stronger correlation and with a more anterior peak in the right hemisphere. Furthermore, there was stronger correlation on the left side, as compared to the right, in the internal part of the lateral fissure around the internal secondary somatosensory cortex. Additionally, there was significant correlation extending from area 46 on the right side. On the left side, we found correlation in middle temporal area.

**Figure 3:**
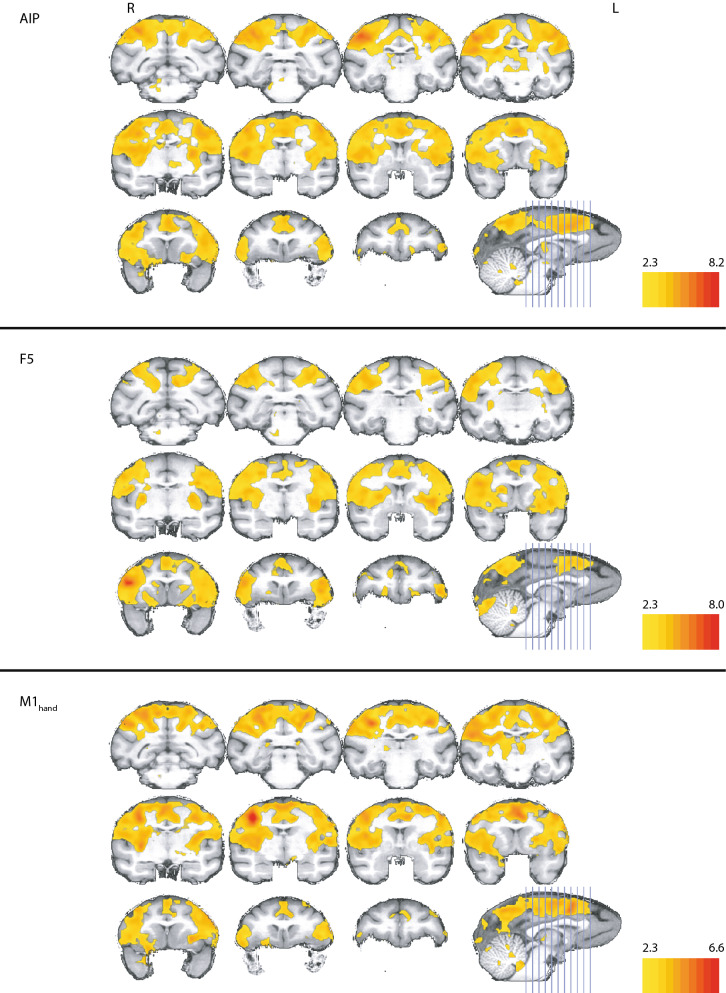
Correlation for AIP, F5 and M1_hand_ in the deep brain structures. Z-statistics thresholded according to the individual z-score color bar. All conventions as in Fig. 2. Y coordinates of the sections are given relative to the F99 reference frame^45^. Sections rendered with MRIcoGL ver. 1.2.20181114 + (https://www.mccauslandcenter.sc.edu/mricrogl/).

Concerning subcortical areas, we found significant correlation in the putamen, external globus pallidus, and the stria medullaris (Fig. 3, top panels). The thalamic nuclei also showed significant correlations, especially the deep mesencephalic nuclei, the body as well as posterior parts of the head of the caudate nucleus, medial geniculate nucleus, and the posterior part of the caudate nucleus.

### Functional connectivity map of ventral premotor cortex (area F5)

When placing seeds in area F5, as part of the ventral premotor cortex, we found strong functional connectivity with the parietal lobe (Fig. 2, middle panels). The posterior border of the correlation map followed the intraparietal sulcus and the lateral fissure, together with some significant correlation at the cuneate gyrus and the superior parietal lobe. Anteriorly, the main cluster extended to the superior arcuate sulcus and at the height of the AC-PC line all the way to the front of the brain. Furthermore, there was a bilateral significant correlation following the principal sulcus, the intraprincipal dimple and the medial orbital sulcus. Cortically, the strongest correlations were located at the right superior temporal sulcus, area 2, the right agranular insular cortex, bilateral in the dysgranular insular cortex, area 45B, opercular parietal area PF (as defined by^50^ roughly equivalent to area 7b in the parcellation of^43^), visual area V3A, and at the anterior part of the intraparietal sulcus and the insular cortex. Significant unilateral correlation was found in the right middle temporal area (V5), whereas in the cingulate cortex correlation was stronger on the left vs. the right hemisphere.

In subcortical structures, significant correlations appeared mostly bilaterally in the putamen, the anterior and posterior limb of the internal capsule, the external capsule and the claustrum, except for the mostly left-lateralized caudate nucleus (Fig. 3, middle panels).

### Functional connectivity map of primary motor cortex

The correlation map of the hand area of M1 also covered the parietal lobe (Fig. 2, bottom panels), with the posterior border of significant correlation marked by the parieto-occipital sulcus, the superior temporal sulcus, and the lateral fissure ventral to the anterior commissure (AC)-posterior commissure (PC) line. Anteriorly, the main cluster of significant correlation reached up to the spur of the arcuate sulcus. In the cingulate sulcus, the cluster extended up to a height of the anterior end of the arcuate sulcus. Here as well, the strongest correlations manifested in the cingulate sulcus, the intraparietal sulcus and the central sulcus. Most of the correlation was bilateral, with the left hemisphere demonstrating higher values than the right one. However, significant correlation was unilateral at the left side of the agranular insular cortex, extending down to the piriform cortex, and on the left side in the lateral area 12, visual area 2, and the medial superior temporal area (MST).

Subcortically, the dorsal part of the thalamic nuclei and part of the fornix, showed significant correlation, as well as the lenticular fasciculus, anterior pulvinar, internal capsule, claustrum, putamen, and the caudate nucleus (Fig. 3, bottom panels). In terms of intensity, the left globus pallidus and the left nucleus accumbens demonstrated stronger correlations than the corresponding right-hemispheric structures.

### Functional connectivity map of area V6A

**Figure 4:**
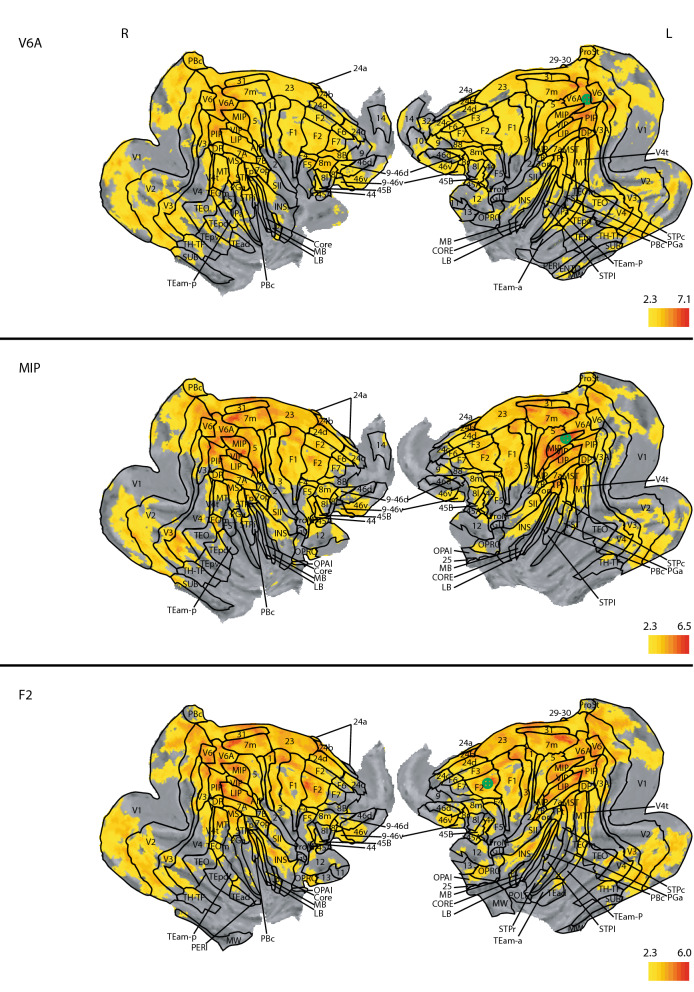
Whole brain correlation of the V6A, MIP and F2 seeds, projected onto the cortical surface. z-statistic maps are FDR corrected and thresholded according to the z-score color bar. Please note that each seed has a different z-score scaling for the projection. Seed placement is marked by the green marker on the left hemisphere. Since the analysis was done on the 3D dataset, the seed placement marking is approximated. All surface renderings (flat maps) were done with CARET v5.65 (https://brainvis.wustl.edu/wiki/index.php/Caret:About)^88^. Cortical area labeling and borders according to^43^.

The correlation map for area V6A covered large areas around the secondary visual cortex and the dorsal half of the parietal and frontal lobe (Fig. 4, top panels). Correlations focused mainly around the cuneus and annectant gyrus and up to the dorsal part of the occipital gyrus, while the lunate sulcus and the calcarine sulcus formed the anterior border of significant correlation. The lingual and the fusiform gyrus also showed prominently correlations. From the posterior end of the lateral sulcus to the anterior end of the intraparietal sulcus, virtually all grey matter was significant, with the fusiform gyrus being the only exception. Furthermore, the cingulate gyrus was significantly correlated along its entire length, the superior temporal gyrus showed significance in its posterior half, whereas at the anterior end of the intraparietal sulcus, only the insula, middle temporal gyrus, and the precentral gyrus showed significant correlation. In the frontal lobe, the superior and middle frontal gyrus showed significant correlations. In general, there was not much deviation from symmetry.

**Figure 5:**
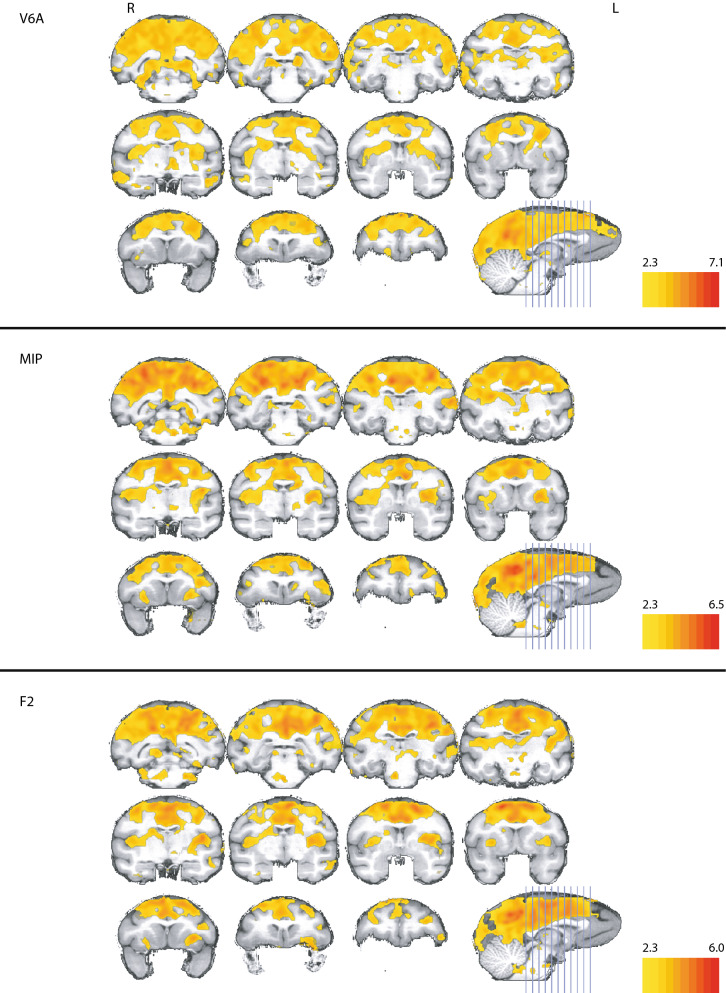
Correlation for V6A, MIP and F2 in the deep brain structures. Z-statistics thresholded according to the individual z-score color bar. All conventions as in Fig. 4. Y coordinates of the sections are given relative to the F99 reference frame^45^. Sections rendered with MRIcoGL ver. 1.2.20181114 + (https://www.mccauslandcenter.sc.edu/mricrogl/).

Subcortical correlations were found mainly in the caudate nucleus, internal capsule, thalamic nuclei, medial pulvinar, and the putamen (Fig. 5, top panels). Results were mainly symmetrical, with exceptions in the frontal part of the claustrum, which had no correlations on the right side, and in the right thalamic nuclei that had stronger and more widespread correlations than in the left.

### Functional connectivity map of MIP

Functional correlation of MIP focused on the visual cortex and the dorsal part of the parietal and frontal lobe, and also prominently featured the cuneus (Fig. 4, middle panels). Significant correlations were also found in the supramarginal and lingual gyrus as well as in the insula. The superior frontal and cingulate gyrus were significant up to their frontal margin. In the frontal lobe, there was strong correlation in area 46 and F2, however, more strongly on the right side than the left. We also saw significant correlations in area 12. All of these correlations were bilateral. Furthermore, there were significant unilateral correlations in the right area 13 and the right somatosensory areas 3b and 2. Posterior to AC, correlations were generally stronger on the left side, while anterior to AC they were stronger on the right side, except for the right fasciolar gyrus. Furthermore, we found significant correlations with the left primary visual cortex.

MIP was also significantly correlated with a number of subcortical structures, most prominently the superior colliculus and the medial and the left oral pulvinar, as well as the putamen and claustrum (Fig. 5, middle panels). Significant correlations were also found in the caudate nucleus anterior to the AC and in the ventral part of the internal capsule and globus pallidus.

### Functional connectivity map of dorsal premotor cortex (area F2)

By placing seeds in dorsal premotor cortex (area F2), we found widespread correlation on the dorsal part of the brain (Fig. 4, bottom panels), covering the superior parietal lobule, the supramarginal gyrus, and dorsal parts of the post- and precentral gyrus. Along the midline and dorsal parts of the cortex we found correlations in the precuneus, cingulate, and lingual gyrus. In the posterior hemisphere, significant correlations appeared along the annectant gyrus and the angular gyrus dorsal to the beginning of the inferior occipital sulcus. In the temporal lobe, correlations were present in the upper third of the middle and superior temporal gyrus, whereas in the frontal lobe strong correlations were present in the superior frontal gyrus and around the principal sulcus. Furthermore, we found strong correlations along the insular cortex.

Subcortically, we saw significant correlations in the superior colliculus, putamen, claustrum, around the pedunculopontine tegmental and cuneiform nuclei, and in the body and parts of the head of the caudate nucleus (Fig. 5, bottom panels). Surprisingly, we found correlations in the right medial globus pallidus and much stronger correlations in the left compared to the right oral pulvinar.

Although all correlation maps had a high degree of symmetry across both hemispheres, most correlations were more widespread on the left, ipsilateral side. In contrast, the correlation map of F2 was more widespread on the contralateral hemisphere. In particular, we found stronger correlations in the right (contralateral) cuneus, the right intraparietal sulcus, in the posterior part of the superior temporal sulcus, and the insular cortex. Correlations in the middle frontal and inferior frontal gyri, however, showed stronger correlation in the left hemisphere.

### Functional connectivity between seed maps

**Figure 6:**
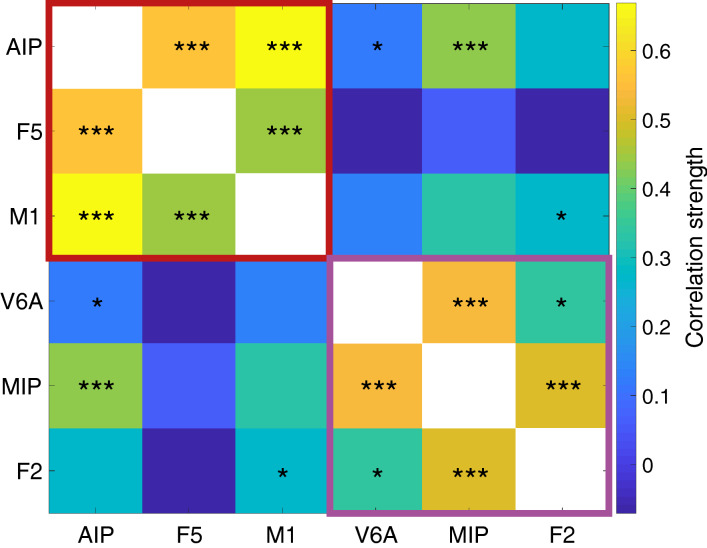
Between seed correlation matrix. Color bar showing the mean correlation between areas over all analyzed animals. Colored frames show the two significant (p = 0.0049) clusters as assessed by optimal modularity^38^. Significance of the correlation between each pair of seed region was tested with a one-sided t test; *p < 0.05 Bonferroni corrected, ***p < 0.01 Bonferroni corrected. Plotted with Matlab Ver. 9.5.0.1067069 (R2018b) Update 4 (https://de.mathworks.com/products/matlab.html).

In addition to individual seed maps, we also calculated the average correlation between the time series of each pair of seed regions across runs (Fig. 6). Strongest significant correlations were found between pairs of seed regions of the same network: AIP/M1_hand_ and AIP/F5 of the grasp-related dorso-ventral network, followed by V6A/MIP and MIP/F2 of the reach-related dorso-medial network. Most correlations between seed region pairs from different networks (AIP/F2, F5/F2, F5/V6A and M1_hand_/V6A) were not significant when tested using a one-sample t-test with Bonferroni correction (p-value: 0.05), with the exception of MIP/AIP, AIP/V6A, and F2/M1_hand_. In particular, the correlation between AIP and MIP was noteworthy, since it was also significant at a higher threshold of 0.01, in contrast to all other correlations across the two designated networks.

**Figure 7:**
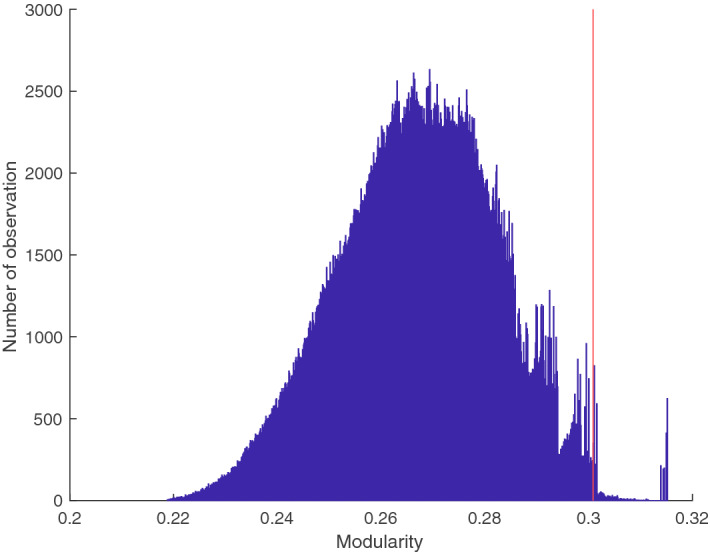
Null distribution of the modularity index of the connectivity matrix. Distribution of the modularity index of 1,000,000 randomly shuffled connectivity matrixes. The red line indicates the modularity index of the original connectivity matrix (0.301). Simulation and plot produced with Matlab Ver. 9.5.0.1067069 (R2018b) Update 4 (https://de.mathworks.com/products/matlab.html).

These findings strongly suggest that the six seed regions belong to two different cortical networks, one containing AIP, M1_hand_ and F5 and a second one including MIP, F2 and V6A. To further test this hypothesis, we computed the optimal modularity of the correlation matrix^38^ using the Brain Connectivity Toolbox^47^. As hypothesized, we found a separation into two networks with a modularity index of 0.301, which was significantly larger than indices produced from surrogate data presuming only a single network (p = 0.0056; matrix shuffling over 1,000,000 random iterations; average modularity index: 0.267, see Fig. 7).

### Evidence for two separate functional networks

**Figure 8:**
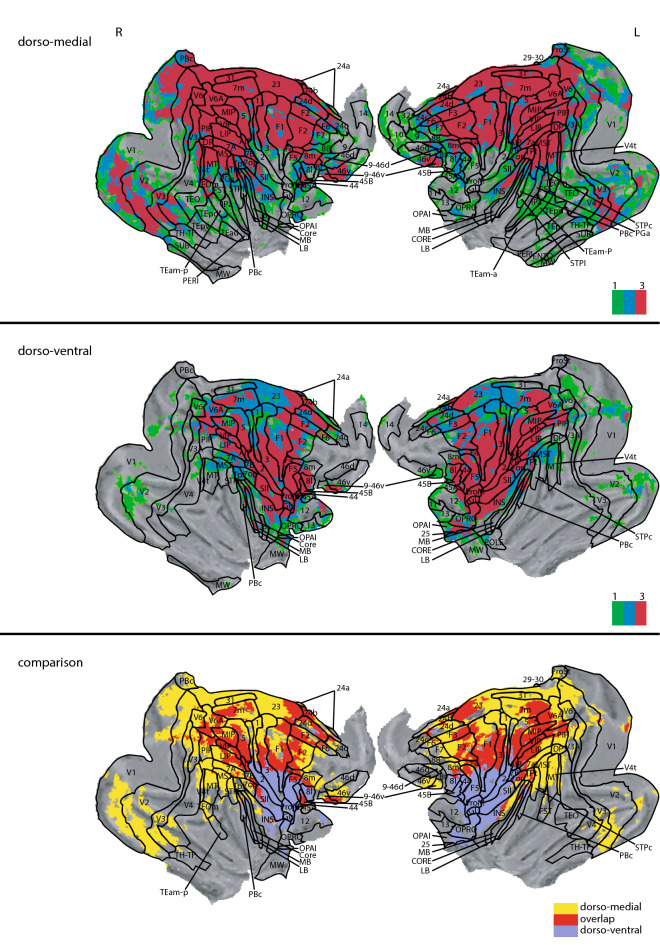
Overlap of the correlation maps of the dorso-medial (top), dorso-ventral (middle), and both networks together (bottom). Overlap was created by projecting the significant voxels of each seed of the respective networks (V6A, MIP and F2 for the dorso-medial and AIP, F5 and M1_hand_ for the dorso-ventral) onto the brain. In the top and middle panel color indicates how often a voxel is significant in the three maps (0–3 times). Voxels that are significant in all three maps of their respective network are considered to show the spatial extend of the network. These voxels are compared in the bottom figure. Here color indicates whether a voxel belongs to the dorso-medial (yellow), dorso-ventral (blue) or both networks (red). All surface renderings (flat maps) were done with CARET v5.65 (https://brainvis.wustl.edu/wiki/index.php/Caret:About)^88^. Cortical area labeling and borders according to^43^.

**Figure 9:**
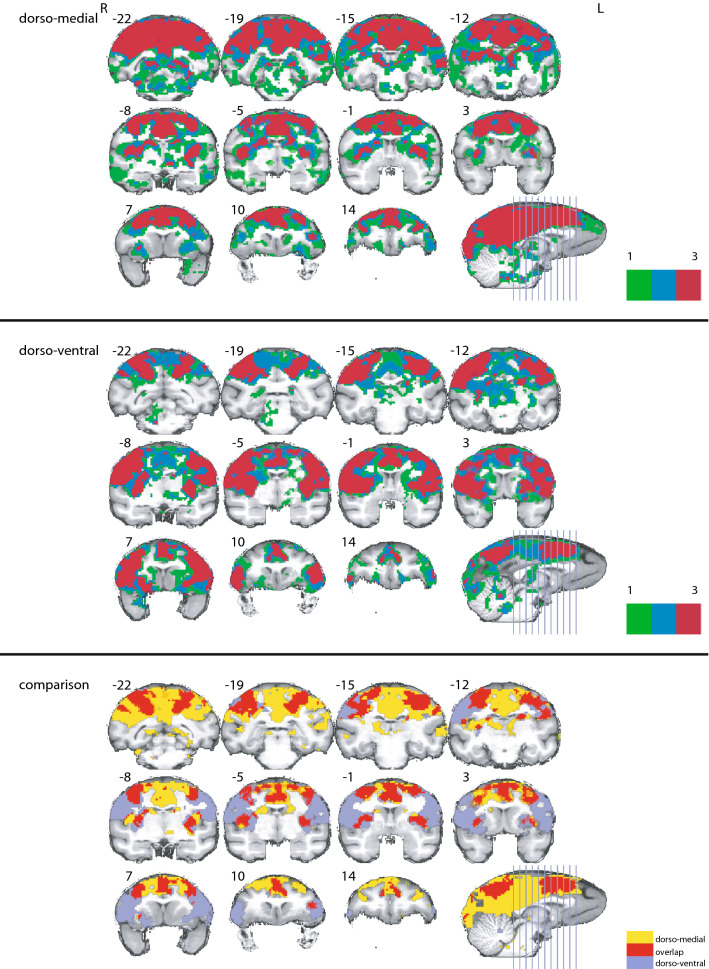
Overlap of the correlation maps of the dorso-medial network seeds (top), the dorso-ventral network seeds (middle) and between both networks (bottom) in the subcortical brain structures. Please note, for ease of comparison, the same color coding is used as in Fig. 8. In the top and middle panel color indicates how often a voxel is significant in the three maps (0–3 times). Bottom panel whether a voxel belongs to the dorso-medial (yellow), dorso-ventral (blue) or both networks (red). Y coordinates of the sections are given relative to the F99 reference frame^45^. Sections rendered with MRIcoGL ver. 1.2.20181114 + (https://www.mccauslandcenter.sc.edu/mricrogl/).

The similarity between the correlation maps of AIP, M1_hand_ and F5 became apparent at first glance (Fig. 2). Figure 8 (middle panels) displays the shared functional correlations between the three dorso-ventral seed areas AIP, M1_hand_, and F5. Areas that appear in the shared dorso-ventral correlation map include the precuneus, the anterior part of the annectant gyrus, the supramarginal, post- and precentral gyrus, and the bilateral intraparietal sulcus (Fig. 8, middle panels). The posterior cingulate gyrus, medial part of the superior frontal gyrus, and the right area 9/46 were also visible in all three maps. In terms of subcortical structures, the putamen and the claustrum showed up in all grasp-related correlation maps (Fig. 9, middle panels).

In the reach-related regions, areas F2, V6A and MIP shared similar patterns in their correlation maps (Fig. 4). The combined correlation map of areas F2, V6A and MIP included large parts of the occipital cortex, starting with the lingual gyrus, predominantly but not exclusively on the right side, the cuneus and precuneus, and the posterior cingulate gyrus (Fig. 8, top panels). We also found large overlaps in the angular gyrus, supramarginal gyrus, superior frontal gyrus and the superior frontal lobule. Along the pre- and postcentral gyrus, the overlap extended laterally from the midline to the end of the intraparietal sulcus. Smaller overlaps were also present along the dorsal aspects of the middle temporal gyrus, the insula, and the principal sulcus. Noteworthy is also area 46, which showed up strongly in both maps. Subcortically, all three correlation maps included the caudate nucleus, putamen, and the claustrum (Fig. 9, top panels).

### Distinct and overlapping areas between the dorso-medial and dorso-ventral functional connectivity maps

Finally, we performed a conjunction analysis across both networks by combining the shared correlation map of AIP, M1_hand_ and F5 for the grasp-related dorso-ventral network and the shared correlation map of V6A, F2 and MIP for the reach-related dorso-medial network. The correlation maps for AIP, M1hand, and F5 (dorso-ventral seeds) were clearly distinct from the correlation maps for V6A, F2, and MIP (dorso-medial seeds), yet showed similar correlation patterns with each other, and vice versa for the dorso-medial seeds. Thus, we refer to the combined correlation maps of AIP, M1_hand_, and F5 as the dorso-ventral network and the combined map of V6A, F2 and MIP as the dorso-medial network (Fig. 8).

When comparing the dorso-medial and dorso-ventral networks, some anatomical structures showed correlations only in one of the two networks while others showed correlations in both (Fig. 8). Although the dorso-ventral network was smaller overall than the dorso-medial one, the lateral part of the precentral and postcentral gyrus was significantly connected only to the grasp-related dorso-ventral network. Similarly, also the inferior ramus of the arcuate sulcus, large parts of the insula, and the anterior part of the claustrum were exclusively correlated with the dorso-ventral network.

In contrast, the cuneus, posterior cingulate gyrus, dorsal part of superior temporal sulcus, left inferior frontal gyrus, and the medial pre- and postcentral gyrus were prominently correlated only with the dorso-medial network. In subcortical regions, the head of the caudate nucleus, the right substantia nigra, and the area around the pulvinar nuclei were exclusively correlated to the dorso-medial network (Fig. 9).

However, a number of areas shared functional connectivity across both networks. These included the precuneus, the superior frontal gyrus, the intraparietal sulcus, and the precentral gyrus medial to the spur of the arcuate sulcus. We also observed large overlap along the intraparietal sulcus, central sulcus, insula and cingulate gyrus. In subcortical regions, the putamen, parts of the internal capsule, and the claustrum shared functional connectivity with both networks.

Together, the conjunction analysis revealed that the dorso-medial and dorso-ventral streams are separated into two distinct functional networks, albeit with some shared functional connectivity across areas in both networks.

## Discussion

Using resting-state fMRI of 10 lightly anesthetized macaque monkeys, we obtained connectivity maps from six key cortical areas in the dorso-ventral and dorso-medial network. Individual correlation maps for the seeds AIP, F5, and the hand area of M1 were combined to form the whole-brain correlation map for the dorso-ventral network, while the correlation maps of the seeds V6A, MIP, and F2 were combined to define the dorso-medial network.

Some brain areas were functionally connected to only one of those networks. They represented sensory inputs and cortical functions related to a specific action (i.e., either reaching or grasping). For example, the dorso-ventral network included somatosensory areas in and around the insular cortex, the precentral opercula (PrCO^51^, which includes parts of areas ProM, SII, GU, 2 and 3 in the parcellation of^43^), gustatory cortex, and the ventral premotor cortex related to hand grasping. In contrast, the dorso-medial network included higher visual areas and areas relevant for arm reaching. Some brain areas, like MIP and AIP, were part of both networks, indicating shared functionality.

In the between-seed correlation, the strong correlation between AIP and MIP is surprising. Although an anatomical connectivity between MIP and AIP has been reported^12^, it was very weak. However, we observed the strongest connections between both networks with high significance between AIP and MIP.

For the dorso-ventral network, we identified the ventral premotor cortex, including areas F4 and F5, and PrCO as exclusively correlated with the grasp-related dorso-ventral network. Areas F4 and F5 represent sensory information and complex motor control signals relevant for planning and execution of hand grasping movements. For example, Hepp-Reymond et al. found in both areas neurons that encode grip force^52^, which is highly relevant for fine object manipulation. Furthermore, pharmacological inactivation of area F5 led to specific grasp deficits, but no reach deficits^16^, hence demonstrating an essential role for hand grasping.

The dorso-ventral network is functionally connected with areas involved in feeding behavior, like the PrCO and the primary gustatory cortex^53^. Especially the correlation with gustatory cortex is interesting. It has been shown by tracer studies that the gustatory cortex is connected to primary motor and sensory areas^54^, but a connection with F5 or AIP, to our knowledge, has not been reported. PrCO is monosynaptically connected to F4 and F5^5^ and has been associated with mechanical and gustatory stimulation of the tongue^55^. Similarly, we found area PF in the inferior parietal lobule to be exclusively correlated with the dorso-ventral network; PF represents mostly orofacial somatosensory as well as biting and feeding responses^56^. These functional connections are well in line with the behavioral relevance of hand grasping for feeding.

Other areas that showed exclusive correlation with the dorso-ventral network were areas 45 and 7, the ventral part of areas 2 and F1, and the granular and dysgranular insula. Most of these areas are involved in somatosensory processing, e.g. as seen in area 2^57^, in line with a notion that a successful grasp requires somatosensory input about the grasp object to tailor hand shape and grip force^58^.

Regions in premotor and sensorimotor cortex that were exclusively correlated with the reach-related dorso-medial network have been previously shown to encode mainly hindlimb movements^59–61^. This could be explained by the fact that arm reaching often also involves the extension of the trunk and hindlimbs to reach a far-away target, which requires the coordination of arms and hindlimbs.

In parietal cortex, most of area V6A was exclusively connected to the dorso-medial network and only small parts were functionally connected with both networks. Area V6A has been extensively investigated with respect to its anatomical connectivity^7,13,62^ and electrophysiological selectivity for reaching as well as grasping behavior^23,33,34,36,63^. However, the presence of selective neural activity does not imply a causal influence, as outlined in the introduction. A selective lesion of V6A produced a reach-to-grasp deficit in macaque monkeys with a strong component of incorrect wrist rotation^64^. However, the animals where still able to close their hand in a functional grasp when the target matched the abnormal wrist orientation. This leaves an open question as to whether the observed reach-to-grasp deficits can be attributed to an uncertainty of the reach process and the wrist orientation, or whether they reflect a true grasp deficit (i.e., a deficit in shaping the hand).

Finally, visual areas V1-V4 also show exclusive functional connectivity with the dorso-medial network. In V1 and V2, functional connectivity is focused mainly on the area that represents peripheral vision^65^, a property that was also true for V6A, whose connectivity to peripheral visual areas has been demonstrated with both tracer^7^ and neurophysiological studies^66^, as well as its encoding of stimulus position in craniotopic coordinates^66^. Reaching requires the position of an object in space, which is predominantly acquired by vision.

Reaching and grasping are closely related motor actions that are often executed together. Tight connectivity between both networks that control these actions is therefore necessary to facilitate a precise and meaningful interaction with the outside world.

We found shared functional connectivity in areas along the intraparietal sulcus (LIP, VIP, V6A, MIP and PIP) that have been shown to provide visual or somatosensory input to premotor areas, e.g., for eye coordination^67–70^. In the supramarginal gyrus, part of the areas PFG and PGop^71^ were significantly correlated with both networks. PFG showed connections to F4 and F5^72^ and has been observed to encode complex somatotopic input not restricted to the hand and arm area^73^. In an extensive mapping of the parietal lobule, Rozzi et al. found sensory and motor neurons similarly distributed to our findings^56^. For example, area PFG contained somatosensory neurons with response fields mainly from the hand and arm, visually responsive neurons to presented objects, and grasp-related motor, peri-personal and mirror neurons. Cortical planning of reaching and grasping actions might require rather similar sensory input, given that both actions need careful sensory coordination.

We also found substantial overlap in the precuneus, whose involvement in reaching and grasping is currently unclear. Although the precuneus has been described as a hub of the default mode network both in humans^74^ and monkeys^75^, the overlap between networks did not appear to correspond with the default mode network. However, tracer studies have shown that the precuneus projects both to the dorsal premotor area F5 and V6A^13,76,77^. Therefore, it is likely that the precuneus correlation is functional rather than caused by the default mode network. Since the precuneus is activated when both hands are coordinated to perform a complex task^78^, we hypothesize that the precuneus plays a role for the coordination of reaching and grasping networks.

Finally, we found shared functional connectivity between networks with the frontal eye field (FEF, area 8 m), which is central for the control of gaze directions^79^ and the integration of sensory information from the dorsal and ventral visual stream^80^. The FEF likely plays an important role for the sensory guidance of arm and hand movements and furthermore for hand–eye coordination.

In conclusion, this study showed that both the dorso-medial and dorso-ventral stream clearly separation at the functional level. The grasp-related dorso-ventral stream is connected more strongly with somatosensory areas, while the reach-related dorso-medial stream has strong connections to visual areas. This separation may be due to arm reaching relying on visual input, while grasping relies more strongly on somatosensory input. This has also been observed in patients who lost their sense of touch; such patients were able to point towards a specific position in space, but had unrecoverable deficits in grasping and manipulating visible objects^81^.

Importantly, we found significant correlations between major areas of both networks, demonstrating that the dorso-medial and dorso-ventral networks are strongly interconnected. Reach and grasp movements are often performed in a coordinated fashion towards a common action goal (e.g., reaching for a branch and climbing the branch or grasping and eating a food item). Connections between these reaching and grasping networks are therefore essential for the spatio-temporal integration and coordination of these actions.

One possible explanation for this interconnection may be adopted from the predictive coding model proposed for motor actions^82–85^. In this model, an efference copy of the actions from an upstream brain area is used by downstream areas to efficiently interpret its input, but this efference copy does not causally influence the actions of the downstream area. This may explain why neural activity in the reach-related dorso-medial stream is correlated with grasping and vice versa.

Finally, our study highlights additional areas that might be involved in the neuronal processing of reaching and grasping actions. For example, the role of the dorsolateral prefrontal cortex (area 9/46) for reaching and grasping has not been well studied but shows extensive functional connectivity with the dorso-medial network. Furthermore, the precentral opercular area and the insular cortex were strongly connected to the dorso-ventral network, as previously demonstrated in tracer studies^86,87^, but without a clear role for the coordination of reaching and grasping. Further electrophysiological studies of these areas may provide new insights into the sensorimotor integration necessary for the successful coordination of reaching and grasping actions.

## Supplementary information

Supplementary Information
