# Supplementary material for: Shared functional connectivity between the dorso-medial and dorso-ventral streams in macaques
Source: Sci Rep. 2020 Oct 29;10:18610. doi: 10.1038/s41598-020-75219-x (PMC7596572; doi:10.1038/s41598-020-75219-x)
Supplement: Supplementary file 1 — Supplementary Information [file 41598_2020_75219_MOESM1_ESM.docx]

Shared functional connectivity between the dorso-medial and dorso-ventral streams in macaques

**Supplemental Information**

Abbreviated Title: Dorso-medial and dorso-ventral stream in macaques

R. Stefan Greulich^1,5,†^, Ramina Adam^2,3^, Stefan Everling^2,4^, Hansjörg Scherberger^1,5, †^

^1^Deutsches Primatenzentrum GmbH, Kellnerweg 4, 37077 Göttingen, Germany

^2^Robarts Research Institute, University of Western Ontario, Canada

^3^Graduate Program in Neuroscience, University of Western Ontario, Canada

^4^Department of Physiology and Pharmacology, University of Western Ontario, Canada

^5^Faculty of Biology and Psychology, Georg-August-University Goettingen, Germany

^†^Corresponding authors. Email: [hscherberger@dpz.eu](mailto:hscherberger@dpz.eu), [sgreulich@dpz.eu](mailto:sgreulich@dpz.eu)

Supplementary Methods: Seed placements

For the seed placements in AIP, F5 and M1_hand_ we used the intraparietal sulcus, the arcuate sulcus, and the central sulcus as guiding landmarks, respectively (see Figure 2 in ^46^). The V6A seed was guided by the parieto-occipital sulcus and the shape of V6A itself. We put the seed as close as possible to the green recording site in Figure 9 of ^34^, as grip encoding cells were reported at this position. MIP was placed according to Figure 1 of ^19^ using the intraparietal sulcus as guidance. F2 was placed using the arcuate and central sulcus, aiming for the diamidino yellow injection site in monkey 3 from ^8^. See table S1 for the coordinates in the F99 reference frame^45^.

Supplementary Table S1

**Seed coordinates.** Coordinates in the F99 reference frame^45^ for seeds in the cortical areas AIP, F5, M1_hand_, V6A, MIP, and F2 in both hemispheres.

| Seed | Coordinates left hemisphere | | | Coordinates right hemisphere | | |
| --- | --- | --- | --- | --- | --- | --- |
|  | X | Y | Z | X | Y | Z |
| AIP | -22 | -14 | 15 | 21 | -14 | 16 |
| F5 | -22 | 6 | 9 | 20 | 6 | 9 |
| M1_hand_ | -17 | -5 | 16 | 17 | -5 | 16 |
| V6A | -6 | -34 | 12 | 6 | -34 | 12 |
| MIP | -5 | -26 | 18 | 5 | -26 | 18 |
| F2 | -9 | 3 | 23 | 11 | 3 | 23 |

## Supplementary Figure S1.


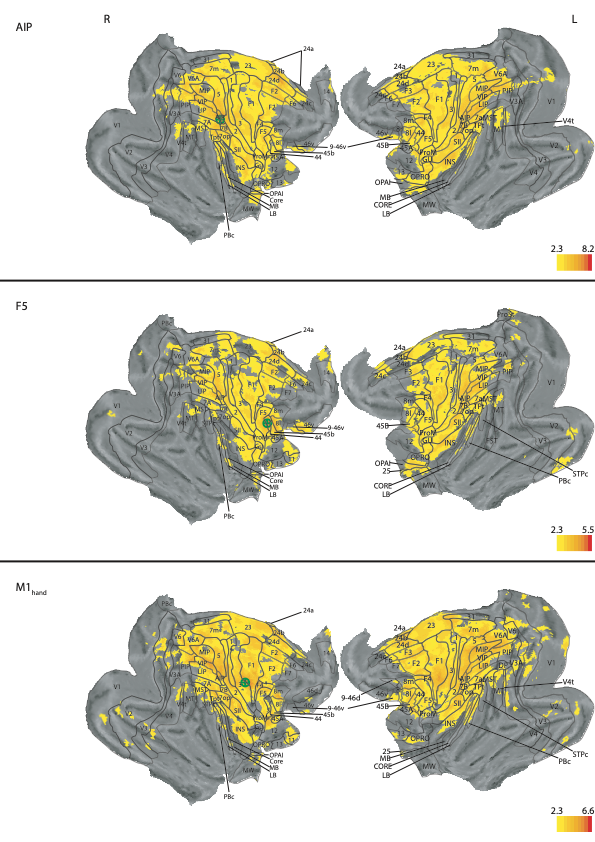


**Cortical correlations of AIP, F5, and M1_hand_ for seeds in the right hemisphere.** Whole brain correlation of the AIP, M1 and F5 seeds, projected onto the cortical surface. z-statistic maps are FDR corrected and thresholded according to the z-score color bar. Seed placement is indicated by green markers on the right hemisphere. Please note that each seed has a different z-score scaling for the projection.

All surface renderings (flat maps) were done with CARET v5.65 (http://brainvis.wustl.edu/wiki/index.php/Caret:About) ^88^. Cortical area labeling and borders according to ^43^.

## Supplementary Figure S2


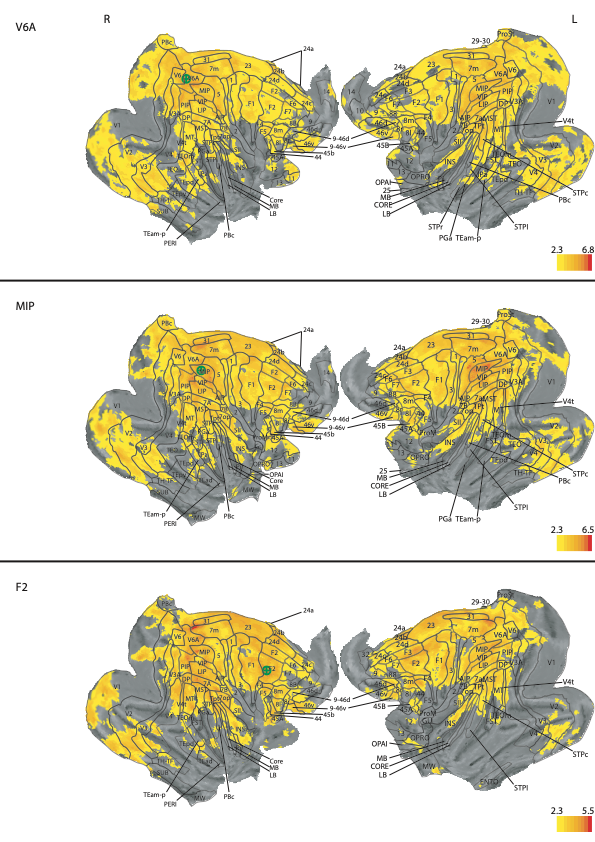


**Cortical correlations of V6A, F2, and MIP for seeds in the right hemisphere.** Whole brain correlation of the V6A, F2 and MIP seeds, projected onto the cortical surface. z-statistic maps are FDR corrected and thresholded according to the z-score color bar. Please note that each seed has a different z-score scaling for the projection. Seed placement is indicated by green markers on the right hemisphere.

All surface renderings (flat maps) were done with CARET v5.65 (http://brainvis.wustl.edu/wiki/index.php/Caret:About) ^88^. Cortical area labeling and borders according to ^43^.
